# Prevalence of Obstructive Sleep Apnea Syndrome in Patients with Resistant Hypertension

**DOI:** 10.3390/jcm15082894

**Published:** 2026-04-10

**Authors:** Songul Ozyurt, Mustafa Taştan, Aziz Gumus, Hatice Beyazal Polat, Neslihan Ozcelik

**Affiliations:** 1 Department of Pulmonology, Faculty of Medicine, Recep Tayyip Erdogan University, 53020 Rize, Türkiye; songul.ozyurt@erdogan.edu.tr (S.O.); aziz.gumus@erdogan.edu.tr (A.G.); neslihan.ozcelik@erdogan.edu.tr (N.O.); 2 Department of Otorhinolaryngology, Faculty of Medicine, Istinye University, 34010 Istanbul, Türkiye; 3 Department of Internal Medicine, Faculty of Medicine, Recep Tayyip Erdogan University, 53020 Rize, Türkiye; hatice.beyazalpolat@erdogan.edu.tr

**Keywords:** obstructive sleep apnea, hypertension, resistant hypertension, polysomnography

## Abstract

**Background**: Obstructive sleep apnea (OSA) is a significant cardiovascular risk factor, frequently co-existing with systemic hypertension. While the association between OSA and blood pressure elevation is well documented, its specific prevalence and impact among patients with resistant hypertension remain a critical area of clinical investigation. Objective: The primary aim of this study was to evaluate the prevalence and severity of obstructive sleep apnea (OSA) specifically within a cohort of patients with resistant hypertension (RHT). Additionally, we sought to identify the clinical and anthropometric factors that distinguish RHT patients with OSA from non-resistant hypertensive and normotensive controls, thereby clarifying the increased hypoxic burden and polysomnographic differences unique to this high-risk population. **Methods**: A total of 300 patients presenting with OSA symptoms were included. Participants were classified into three groups: Group 0 (*n* = 100), normotensive individuals; Group 1 (*n* = 100), non-resistant hypertension; and Group 2 (*n* = 100), confirmed resistant hypertension. Standard overnight polysomnography (PSG) was performed on all participants. Demographic data, comorbidities, anthropometric measurements, and PSG parameters were recorded and compared across groups. **Results**: Of the subjects, 50.3% were female (sex), and the mean age was 49.5 ± 12.3 years. Patients with RHT (Group 2) were significantly older and had a higher prevalence of diabetes mellitus. OSA prevalence was 94% in Group 2 (37% severe), 89% in Group 1 (22% severe), and 74% in Group 0 (only 2% severe). In PSG analysis, AHI and ODI values were significantly higher in hypertensive groups (Groups 1 and 2) compared to normotensive individuals (Group 0), while minimum and mean oxygen saturations were significantly lower. **Conclusions**: OSA is both more prevalent and more severe in patients with resistant hypertension. Furthermore, hypertensive OSA patients are characterized by an increased hypoxic load compared to normotensives. Systematic investigation and detailed polysomnographic evaluation of OSA are of paramount importance in hypertensive individuals, particularly those with resistant hypertension.

## 1. Introduction

Resistant hypertension (RHT) is defined as blood pressure that remains above target levels despite appropriate lifestyle measures and the concurrent use of at least three antihypertensive agents, including a diuretic, at their maximum tolerated doses. Patients whose blood pressure is controlled but require four or more medications are also categorized within this group [1,2,3]. With a reported prevalence ranging from 2% to 40%, RHT represents a significant clinical challenge associated with increased target organ damage, cardiovascular events, and a substantial burden on the healthcare system [3,4].

It has been demonstrated that patients with resistant hypertension face a significantly elevated risk of mortality, myocardial infarction, heart failure, stroke, and chronic kidney disease [4,5]. Consequently, the systematic investigation of secondary causes in RHT is one of the fundamental pillars of clinical management [6,7].

Growing evidence suggests that obstructive sleep apnea (OSA) is one of the most common and potentially reversible causes of resistant hypertension. OSA-related intermittent hypoxia impairs blood pressure regulation through sympathetic activation, stimulation of the renin–angiotensin–aldosterone system (RAAS), and endothelial dysfunction [8,9]. While the prevalence of OSA in hypertensive patients ranges from 37% to 56%, it reaches 70–83% in those with resistant hypertension [8,10,11,12]. Significant correlations have been reported between OSA severity, blood pressure levels, and the requirement for antihypertensive medications; furthermore, continuous positive airway pressure (CPAP) therapy has been shown to improve blood pressure control (8,12). Systematic reviews and meta-analyses also indicate that the presence of OSA increases the risk of resistant hypertension [13]. However, comparative studies validated by polysomnography (PSG) investigating the relationship between OSA severity and blood pressure control remain limited.

The primary aim of this study was to evaluate the prevalence and severity of obstructive sleep apnea (OSA) specifically within a cohort of patients with resistant hypertension (RHT). Additionally, we sought to identify the clinical and anthropometric factors that distinguish RHT patients with OSA from non-resistant hypertensive and normotensive controls, thereby clarifying the increased hypoxic burden and polysomnographic differences unique to this high-risk population.

## 2. Materials and Methods

### 2.1. Study Design and Participants

This prospective observational study included a total of 300 participants, divided into three groups: Group 2 (Resistant Hypertension, *n* = 100), Group 1 (Controlled Hypertension with one or two medications, *n* = 100), and Group 0 (Normotensive, *n* = 100). Groups 0 and 1 consisted of consecutive patients presenting with symptoms of excessive daytime sleepiness, snoring, and witnessed apnea who underwent polysomnography (PSG). Following Ethics Committee approval, patient recruitment was conducted between 9 January 2025, and 20 December 2025. All participants underwent overnight PSG for the diagnosis of obstructive sleep apnea (OSA). Demographic data comorbidities, smoking status, Epworth Sleepiness Scale (ESS) scores, waist and neck circumferences, laboratory findings, and PSG parameters were recorded.

Inclusion Criteria: Volunteer patients aged 18 and over with technically adequate PSG recordings and who provided written informed consent.

Exclusion Criteria:

- Previous diagnosis or treatment for OSA.
- Advanced chronic lung disease.
- Decompensated heart failure (NYHA Class III–IV).
- End-stage renal disease (eGFR < 15 mL/min/1.73 m^2^).
- Active infection, malignancy, or multi-organ failure.
- Pregnancy.
- Impaired cognitive function.
- Inability to undergo a sleep study or provide informed consent.
- Secondary etiologies of hypertension, such as primary hyperaldosteronism: While we did not systematically measure plasma aldosterone levels, these causes were excluded through a careful review of each patient’s medical history, physical exams, and routine lab results (e.g., persistent low potassium or metabolic alkalosis).
- Non-adherence to antihypertensive medication therapy.

This study was conducted in accordance with the Declaration of Helsinki and approved by the institutional ethics committee (Approval No: 2025/05).

### 2.2. Polysomnography

Sleep monitoring was performed using a polysomnography-based respiratory monitoring system (Natus^®^ Brain Monitor PSG, Natus Medical Incorporated, Oakville, ON, Canada). Parameters obtained included the Apnea–Hypopnea Index (AHI), mean oxygen saturation (MSaO_2_), lowest oxygen saturation (LSaO_2_), and Oxygen Desaturation Index (ODI). Apnea was defined as a ≥90% reduction in oronasal airflow for at least 10 s. Hypopnea was defined as a ≥30% reduction in airflow for ≥10 s accompanied by ≥4% oxygen desaturation, or a ≥50% reduction accompanied by ≥3% desaturation [14]. OSA diagnosis and severity were determined according to the 2012 American Academy of Sleep Medicine (AASM) criteria [15].

### 2.3. Diagnosis of Resistant Hypertension

Resistant hypertension was evaluated by an internal medicine specialist according to current international guidelines. It was defined as blood pressure remaining above target levels despite the use of three different classes of antihypertensive drugs at maximally tolerated doses (including a diuretic) or requiring four or more medications for blood pressure control [1]. Blood pressure was measured using validated automated devices following guideline recommendations; the average of two measurements taken 1–2 min apart after at least 5 min of rest in a seated position was recorded. Phenotypic evaluation and auscultation of the heart and renal arteries were performed for all patients. Laboratory samples were collected following an 8 h overnight fast. Fasting plasma glucose, lipid profile, electrolytes, serum creatinine, estimated glomerular filtration rate (eGFR) calculated via the CKD-EPI formula, and urinalysis were analyzed.

### 2.4. Clinical and Anthropometric Evaluation

Demographic data, lifestyle characteristics, and comorbidities were recorded. Anthropometric measurements, including body mass index (BMI), neck circumference, and waist circumference, were evaluated using standardized methods.

### 2.5. Dataset Composition and Variables

The dataset comprised demographic, clinical, anthropometric, laboratory, and PSG data from 300 patients. Variables included age, sex, smoking history, presence of hypertension/RHT, cardiovascular disease, diabetes mellitus, and chronic airway disease. Laboratory parameters covered inflammatory markers, hematological indices, liver function tests, and lipid profiles. Subjective daytime sleepiness was assessed using the Epworth Sleepiness Scale.

### 2.6. Statistical Methods

Descriptive statistical analyses were performed using IBM-SPSS (version 27; SPSS Inc. Chicago, IL, USA). Parametric variables were expressed as mean ± standard deviation, non-parametric variables as median (interquartile range), and categorical variables as percentages. For the comparison of the three groups, One-Way ANOVA was used for normally distributed variables, while the Kruskal–Wallis test was applied to non-normally distributed data. Categorical variables were compared using the Chi-square test. A *p*-value of <0.05 was considered statistically significant.

## 3. Results

Of the total 300 patients presenting with symptoms suggestive of OSA and undergoing PSG, 151 (50.3%) were female. The mean age of the study population was 49.5 ± 12.3 years. A history of smoking was present in 92 (30.7%) patients. Regarding comorbidities, 12 (4%) had chronic respiratory disease, 21 (7%) had coronary artery disease (CAD), and 35 (11.7%) had diabetes mellitus (DM). Participants were categorized as Group 0 (Normotensives), Group 1 (Non-resistant Hypertensives), and Group 2 (Resistant Hypertensives).

Patients in Group 2 were found to be significantly older than those in Group 0 and Group 1 (*p* = 0.001 and *p* = 0.048, respectively). Additionally, Group 1 patients were significantly older than Group 0 (*p* = 0.001). The prevalence of DM was significantly higher in Group 2 compared to Groups 1 and 0; similarly, Group 1 had a significantly higher prevalence of DM compared to Group 0 (*p* = 0.041, *p* = 0.001, and *p* = 0.001, respectively).

**Figure 1:**
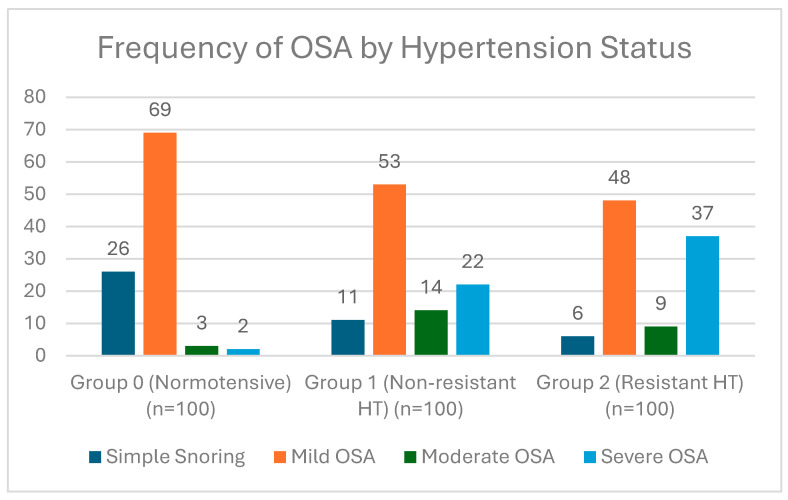
Distribution of OSA Severity Based on Hypertension Status.

Among the 100 cases in Group 2, OSA was identified in 94% of the patients, of whom 37 (37%) had severe OSA, 9 (9%) had moderate OSA, and 48 (48%) had mild OSA. In Group 1, the prevalence of OSA was 89%, with 22 patients (22%) diagnosed with severe OSA. In Group 0, severe OSA was detected in only 2 individuals, and 26% of this group had no OSA (Figure 1).

**Figure 2:**
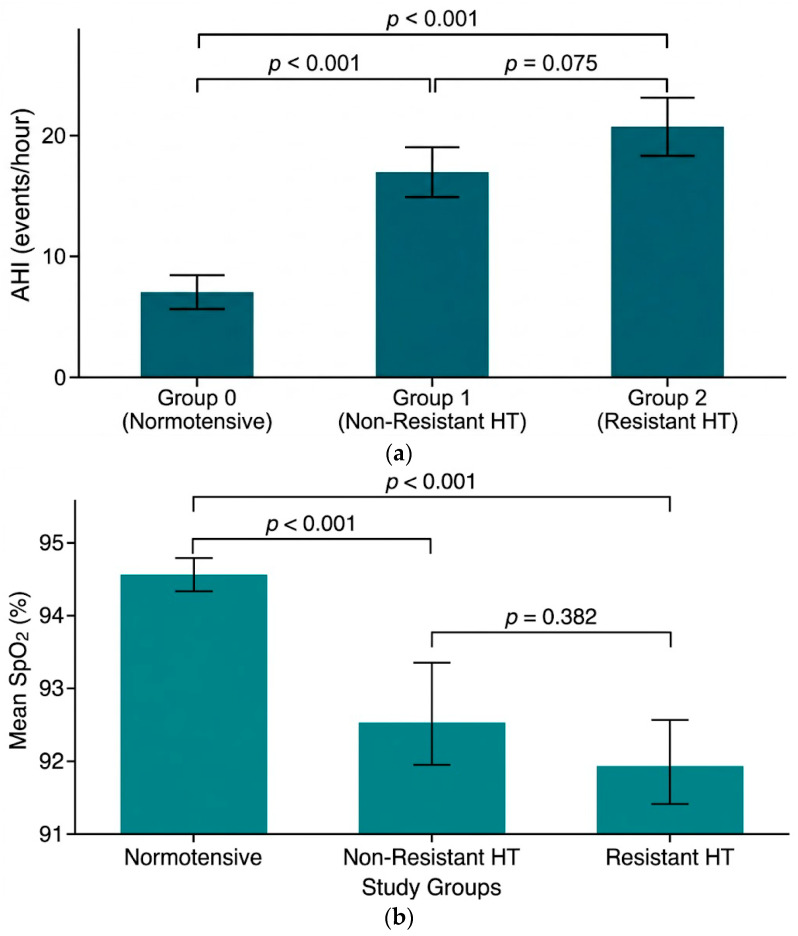
(**a**) Representation of the differences in AHI values among groups categorized by hypertension status using an error bar plot. (**b**) Mean oxygen saturation percentages across hypertension status groups shown with an error bar graph.

All groups were compared in terms of demographic characteristics, comorbidities, anthropometric measurements, laboratory findings, and OSA parameters (Table 1 and Table 2). In the overall cohort, normotensive patients were significantly younger than hypertensive patients. AHI (Apnea–Hypopnea Index) and ODI (Oxygen Desaturation Index) values were significantly higher in Group 2 and Group 1 compared to Group 0 (*p* = 0.001), while mean and minimum oxygen saturation levels were significantly lower (*p* = 0.001). Although AHI values were higher in Group 2 compared to Group 1, this difference did not reach statistical significance (*p* = 0.075) (Figure 2a,b).

## 4. Discussion

In this prospective study, the prevalence of obstructive sleep apnea (OSA) was shown to be significantly higher in patients with resistant hypertension (RHT) compared to non-resistant hypertensive and normotensive individuals. The identification of an exceptionally high OSA prevalence of 94% in the resistant hypertension group strongly supports the role of OSA as a common and potentially reversible secondary cause in this patient population.

The literature reports that OSA prevalence in patients with resistant hypertension ranges between 70% and 83%, with rates increasing further in studies utilizing systematic polysomnography [12,16,17]. Pedrosa et al. emphasized that OSA is the most frequently identified secondary cause in the resistant hypertension population [12]. Furthermore, a study involving 422 patients with resistant hypertension reported an OSA prevalence of 82.2%, with 55.5% of these patients categorized in the moderate-to-severe OSA group [18]. While our findings confirm these high prevalence rates, they also suggest that OSA may almost be the rule rather than the exception in patients with resistant hypertension. OSA is known to contribute to impaired blood pressure regulation through increased sympathetic activity, decreased baroreflex sensitivity, endothelial dysfunction, systemic inflammation, and alterations in salt and water metabolism [8,9,12]. The sharing of common risk factors, such as obesity, between resistant hypertension and OSA is another significant factor explaining this association [12,19].

Another crucial finding of our study is that OSA is not only more frequent, but also more severe in patients with resistant hypertension. The 37% rate of severe OSA in this group suggests a significant relationship between OSA severity and the resistant hypertension phenotype. Previous studies have proposed that higher AHI values in RHT patients may be associated with increased nocturnal fluid shift from the legs to the neck region, where rostral fluid displacement plays a role in upper airway obstruction [20]. While we explored the role of nocturnal rostral fluid shift in airway obstruction, it is important to consider that aldosterone excess may act as an upstream driver for this process by increasing sodium and water retention. Although hormonal assays were not part of our protocol, this physiological interplay remains a plausible contributor to the severity of OSA in our resistant hypertension group. In our study, AHI values in the resistant hypertension group were significantly higher than in the normotensive group; although they were also higher compared to the non-resistant hypertension group, this difference did not reach statistical significance (*p* = 0.075). This may be related to the sample size; however, it remains clinically significant. Indeed, high AHI values have been shown to increase the risk of mortal and non-mortal cardiovascular events, whereas effective OSA treatment significantly reduces these risks [21].

Repeated upper airway obstructions in OSA impair ventilation, leading to hypoxemia. Therefore, the AHI, which only reflects the number of obstructive events per hour, may not always fully capture the physiological burden. Observational studies have reported that indicators of hypoxic load, such as the duration of time oxygen saturation remains below 90% during the night, are stronger predictors of cardiovascular disease and all-cause mortality compared to the AHI [22]. Accordingly, oxygenation parameters were analyzed in detail in our study. The significantly higher Oxygen Desaturation Index (ODI) values and markedly lower minimum and mean oxygen saturations in both hypertensive groups compared to the normotensive group support the central role of the hypoxic load concept in the OSA-hypertension relationship. The lack of a statistically significant difference in these parameters between the resistant and non-resistant hypertension groups may be attributed to the limited number of patients. Nevertheless, the trend toward higher ODI values and lower oxygen saturations in the resistant hypertension group is clinically noteworthy. Intermittent hypoxia-induced sympathetic activation, stimulation of the renin–angiotensin–aldosterone system, oxidative stress, and endothelial dysfunction are proposed as the fundamental mechanisms underlying persistent blood pressure elevation, particularly in patients with resistant hypertension [23].

The close relationship between OSA and insulin resistance, glucose intolerance, and metabolic syndrome is well established [24]. In our study, the significantly higher prevalence of diabetes mellitus in the resistant hypertension group compared to other groups is consistent with the literature [25]. The inflammatory response and adipokine imbalance triggered by intermittent hypoxia may contribute to the deepening of metabolic disturbances and the evolution of hypertension into a more resistant form [24,25]. These findings point toward a complex clinical picture where resistant hypertension, OSA, and metabolic comorbidities frequently coexist [26].

The relatively high prevalence of OSA found in normotensive individuals (74%) is related to the inclusion of patients already presenting with OSA symptoms; however, the low rate of severe OSA in this group is clinically remarkable. Literature reports that normotensive OSA patients typically present with a milder disease spectrum and lower hypoxic load [27]. Nonetheless, it has been shown that normotensive OSA patients at baseline face an increased risk of developing hypertension in the future; even minimally elevated AHI has been shown to increase the risk of developing hypertension approximately threefold over a 4-year follow-up [28]. This finding suggests that untreated OSA may be a significant cause of uncontrollable hypertension in the long term.

The clinical management of patients with RHT and OSA is entering a new therapeutic era with the development of novel aldosterone synthase inhibitors. Historically, Phase 3 trials for these agents have largely excluded patients with both resistant hypertension and OSA. However, our finding of a 94% OSA prevalence in the RHT group underscores the urgent need to include this population in clinical drug development. Current evidence, such as the lorundrostat development programme, is now shifting focus toward this underserved group. Given the strong pathophysiological rationale for targeting aldosterone to reduce both blood pressure and nocturnal fluid-driven airway obstruction, these novel therapies could represent a landmark shift in the treatment of this high-risk population [29].

### Strengths and Limitations

The limitations of this study include its single-center and cross-sectional design, which limits the generalizability of the findings. The inclusion of individuals with OSA symptoms may have led to a relatively high OSA prevalence, particularly in the normotensive group, and differences in age and metabolic comorbidities between groups may pose potential confounding effects. Furthermore, while metabolic data were collected, the lack of systematic HbA1c measurements for all participants may have limited our ability to identify undiagnosed prediabetes or diabetes cases, potentially accounting for the lower-than-expected prevalence of diabetes in the obese cohort. Additionally, detailed cardiovascular characterization—including atrial fibrillation subtypes, comprehensive echocardiographic parameters (such as HFpEF, LVH, and LA enlargement), and specific cardiac biomarkers (NT-proBNP, hs-TnT)—was not predefined as a study outcome and was obtained from existing medical records. This may have limited the assessment of the full clinical impact of cardiovascular comorbidities in our study population.

Furthermore, the effects of OSA treatments, particularly CPAP, on blood pressure and polysomnographic parameters were not evaluated. The lack of systematic measurement for plasma aldosterone and renin levels is another limitation. Given that aldosterone can drive both blood pressure resistance and upper airway collapse through fluid redistribution, future studies incorporating these hormonal evaluations would provide a more comprehensive understanding of the OSA-hypertension relationship. Finally, the use of the Epworth Sleepiness Scale (ESS) instead of more comprehensive screening tools like the STOP-BANG score might have limited the multi-dimensional risk stratification of our participants.

Conversely, the methodological strengths of this study include the comparison of three groups (resistant hypertensive, non-resistant hypertensive, and normotensive individuals) through prospective and consecutive patient recruitment. Additional strengths include the verification of resistant hypertension diagnosis by an internal medicine specialist in accordance with current guidelines, including the assessment of triple-drug regimens and clinical adherence, and the application of standard full-night polysomnography to all participants in the same center.

## 5. Conclusions

Patients with resistant hypertension are associated with a high risk of cardiovascular and renal morbidity, representing a significant public health burden. This study demonstrates that obstructive sleep apnea (OSA) manifests with a high prevalence and increased severity in patients with resistant hypertension. Our findings support that systematic screening and effective treatment of OSA, particularly in individuals with resistant hypertension, should be a fundamental clinical strategy in reducing long-term cardiovascular risk.

## Figures and Tables

**Table 1:** Comparison of Demographic and Anthropometric Characteristics Among the Study Groups.

Variables	Group 0 (Normotensive) (*n* = 100)	Group 1 (Non-Resistant HT) (*n* = 100)	Group 2 (Resistant HT) (*n* = 100)	*p* Value
Age (years)	40.5 ± 12.1	52.3 ± 8.9	55.8 ± 10.1	
Sex (F/M)	56/44	41/59	54/46	0.07
Smoking, *n* (%)	37 (37%)	28 (28%)	27 (27%)	0.24
Smoking (pack-year)	0 (0–10)	0 (0–8.7)	0 (0–5)	0.465
Chronic Respiratory Disease, *n* (%)	4 (4%)	2 (2%)	6 (6%)	0.353
Diabetes Mellitus, *n* (%)	0 (0%)	12 (12%)	23 (23%)	
Coronary Artery Disease, *n* (%)	0 (0%)	10 (10%)	11 (11%)	
Height (cm)	167 ± 9	169 ± 7	165 ± 8	0.127 (a), 0.503 (b), 0.007 (c)
Weight (kg)	89 ± 20	96 ± 18	95 ± 17	0.091 (a), 0.045 (b), 0.955 (c)
BMI (kg/m^2^)	32.1 ± 6.9	34.2 ± 6.1	35.2 ± 6.4	0.003 (a), 0.050 (b), 0.577 (c)
Waist Circumference (cm)	103 ± 17	112 ± 14	111 ± 15	0.004 (a), 0.002 (b), 0.966 (c)
Neck Circumference (cm)	38.6 ± 4.2	40.6 ± 3.8	40.6 ± 3.7	

Abbreviations: HT: Hypertension; BMI: Body Mass Index. Notes: Data are presented as mean ± standard deviation, median (interquartile range [IQR]), or number and percentage (%). a: Comparison between Group 2 and Group 0. b: Comparison between Group 1 and Group 0. c: Comparison between Group 2 and Group 1.

**Table 2:** Comparison of Clinical and Polysomnographic Characteristics Among the Study Groups.

Variables	Group 0 (Normotensive) (*n* = 100)	Group 1 (Non-Resistant HT) (*n* = 100)	Group 2 (Resistant HT) (*n* = 100)	*p* Value
Epworth Sleepiness Scale Score	4 (2–7.7)	4.5 (2–8)	4 (2–7.8)	0.709
AHI (events/hour)	6.7 (4.9–9.6)	10.3 (6.6–29.4)	11 (8–34)	
ODI (events/hour)	2.9 (0.7–6.7)	8.2 (3.6–19.3)	10 (4–22)	
Mean SpO2 (%)	94.5 ± 1.5	92.5 ± 3.2	91.9 ± 3.4	
Minimum SpO2 (%)	88.0 ± 6.9	82.5 ± 8.9	82.8 ± 7.9	
CRP (mg/L)	4.9 (2–7.8)	2.9 (1.9–5.0)	3.0 (1.9–6.6)	0.243
Leukocyte Count (/µL)	7425 ± 1828	7441 ± 1836	7533 ± 1983	0.884
Neutrophil Count (/µL)	4266 ± 1512	4110 ± 1264	4447 ± 1559	0.268
Lymphocyte Count (/µL)	2526 ± 635	2677 ± 783	2457 ± 749	0.093
Platelet Count (×10^3^/µL)	276 ± 73	277 ± 62	273 ± 61	0.892
RDW (%)	13.6 ± 1.1	13.7 ± 1.0	13.8 ± 0.8	0.753
MPV (fL)	10.7 ± 1.3	10.5 ± 1.1	10.6 ± 1.2	0.836
ALT (U/L)	23 (16–35)	24 (17–37)	19 (14–27)	0.046 (a), 0.394 (b), 0.003 (c)
AST (U/L)	21 (17–25)	22 (18–28)	19 (17–25)	0.680 (a), 0.068 (b), 0.011 (c)
GGT (U/L)	22 (16–32)	25 (20–39)	23 (18–32)	0.699 (a), 0.006 (b), 0.020 (c)
Total Bilirubin (mg/dL)	0.59 (0.46–0.81)	0.6 (0.47–0.77)	0.57 (0.44–0.78)	0.971
Glucose (mg/dL)	95 ± 17	101 ± 19	107 ± 37	0.003 (a), 0.161 (b), 0.283 (c)
LDL Cholesterol (mg/dL)	135 ± 40	136 ± 37	129 ± 41	0.376
HDL Cholesterol (mg/dL)	48 ± 13	50 ± 11	53 ± 14	0.015 (a), 0.734 (b), 0.103 (c)
Total Cholesterol (mg/dL)	210 ± 48	218 ± 44	213 ± 45	0.445
Triglycerides (mg/dL)	131 (93–192)	152 (104–197)	155 (109–206)	0.104

Abbreviations: HT: Hypertension; AHI: Apnea–Hypopnea Index; ODI: Oxygen Desaturation Index; SpO2: Peripheral Oxygen Saturation; CRP: C-reactive Protein; RDW: Red Cell Distribution Width; MPV: Mean Platelet Volume; ALT: Alanine Aminotransferase; AST: Aspartate Aminotransferase; GGT: Gamma-Glutamyl Transferase; LDL: Low-Density Lipoprotein; HDL: High-Density Lipoprotein. Notes: Data are presented as mean ± standard deviation, median (interquartile range [IQR]), or number and percentage (%). a: Comparison between Group 2 and Group 0. b: Comparison between Group 1 and Group 0. c: Comparison between Group 2 and Group 1.

## Data Availability

The data presented in this study are available on request from the corresponding author. The data are not publicly available due to ethical and privacy restrictions.
